# mRNA Translation Is Dynamically Regulated to Instruct Stem Cell Fate

**DOI:** 10.3389/fmolb.2022.863885

**Published:** 2022-03-31

**Authors:** Ruoxu Wang, Marc Amoyel

**Affiliations:** Department of Cell and Developmental Biology, University College London, London, United Kingdom

**Keywords:** stem cell, self-renewal, differentiation, translation, protein synthesis, mTOR, eIF2 kinase, ribosome biogenesis

## Abstract

Stem cells preserve tissue homeostasis by replacing the cells lost through damage or natural turnover. Thus, stem cells and their daughters can adopt two identities, characterized by different programs of gene expression and metabolic activity. The composition and regulation of these programs have been extensively studied, particularly by identifying transcription factor networks that define cellular identity and the epigenetic changes that underlie the progressive restriction in gene expression potential. However, there is increasing evidence that post-transcriptional mechanisms influence gene expression in stem cells and their progeny, in particular through the control of mRNA translation. Here, we review the described roles of translational regulation in controlling all aspects of stem cell biology, from the decision to enter or exit quiescence to maintaining self-renewal and promoting differentiation. We focus on mechanisms controlling global translation rates in cells, mTOR signaling, eIF2ɑ phosphorylation, and ribosome biogenesis and how they allow stem cells to rapidly change their gene expression in response to tissue needs or environmental changes. These studies emphasize that translation acts as an additional layer of control in regulating gene expression in stem cells and that understanding this regulation is critical to gaining a full understanding of the mechanisms that underlie fate decisions in stem cells.

## Introduction

Stem cells share the unique property of being able to both self-renew and differentiate, generating progeny with specialized functions. Nonetheless, stem cells encompass a wide variety of cells with a broad range of behaviors, from multipotent embryonic stem cells, which give rise to all cell types in an embryo, to lineage-restricted adult stem cells. For instance, in some mammalian tissues such as the blood, muscle, or brain, stem cells are mostly quiescent and proliferate only when activated by environmental signals, while in other tissues such as the intestine or epidermis, stem cells are highly proliferative to maintain tissue integrity despite continued turnover of differentiated cells. Even in those tissues, differentiation or proliferation can be modulated in response to stimuli from dying cells, or systemic signals including nutrition. Thus, adult stem cells are capable of rapidly altering their behavior and fate in response to the needs of the tissue or the organism, emphasizing the flexibility in their gene expression programs.

How gene expression programs controlling quiescence, proliferation, self-renewal, and differentiation can be both stable and plastic is the subject of much study, often focusing on understanding the transcriptional networks that maintain cell identity and the inputs that destabilize these networks and allow cells to change fate. Our understanding of these networks has grown and is continually being refined, showing that various stable network states exist and explaining transitions between these states (Kim et al., 2008; Moignard et al., 2013; Theunissen and Jaenisch, 2017; Kim et al., 2020; Sagner et al., 2021). Furthermore, we have gained considerable understanding of the epigenetic changes that reinforce these transcriptional changes and ensure that stem cells maintain plasticity in gene expression while differentiating cells gradually become restricted in potential (Lunyak and Rosenfeld, 2008; Ohbo and Tomizawa, 2015; Theunissen and Jaenisch, 2017; Ding et al., 2021).

However, technological advances enabling the comparison of the proteins in cells with their transcriptome led to the discovery that the two are often poorly correlated and that changes in the proteome can occur without accompanying transcriptional changes (Unwin et al., 2006; de Sousa Abreu et al., 2009; Lu et al., 2009; Maier et al., 2009; Schwanhausser et al., 2011), indicating that there are additional layers of regulation of gene expression beyond transcription. This mismatch has been described in many cell types, including stem cells (Lu et al., 2009; Ingolia et al., 2011; Baser et al., 2019; Habowski et al., 2020; Spevak et al., 2020), suggesting that post-transcriptional control of gene expression is common. The protein content of a cell depends on both synthesis and degradation: work describing extensive links between protein degradation and stem cell fate has been reviewed elsewhere (Strikoudis et al., 2014; Suresh et al., 2016; Yan et al., 2020); here, we will focus on the mechanisms affecting stem cell fate through the regulation of protein synthesis.

### Bulk Translation Rates Change During Stem Cell Activation and Differentiation

Until recently, precise measurement of translation rates was mostly restricted to cell culture models where newly synthesized proteins could be labeled by providing a pulse of radioactive amino acids. For instance, in *ex vivo* cultures, differentiating murine embryonic stem cells (mESCs) into structures known as embryoid bodies resulted in a ∼2-fold increase in their translation rate, as indicated by [^35^S] methionine incorporation (Sampath et al., 2008). Consistently, embryoid bodies display an increased content of the Golgi apparatus and rough endoplasmic reticulum (ER) and an increased proportion of polysomes (multiple ribosomes bound to the same mRNA), indicating higher rates of protein synthesis. Similarly, cultured human embryonic stem cells (hESCs) show immature Golgi and rough ER and much lower translation rates than differentiated derivatives (Easley et al., 2010).

**FIGURE 1:**
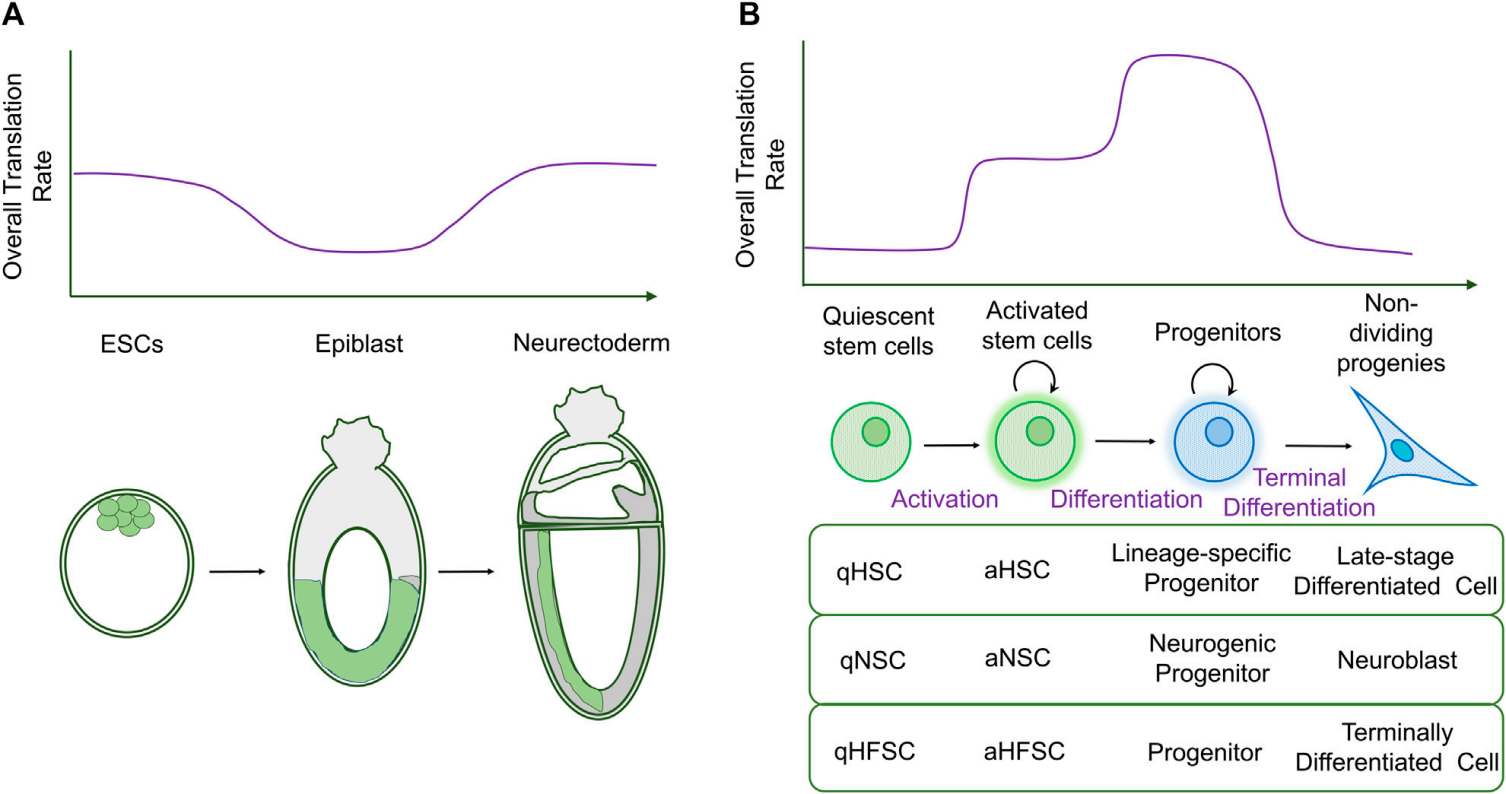
Changes in global translation during stem cell differentiation. **(A)** Diagram schematizing bulk translation rates during early mammalian embryogenesis (top), with stages shown later. Bulk translation rates are initially high in ESCs and decrease during differentiation into the epiblast. Global translation levels rise again during neurectodermal development. **(B)** In adult stem cells, quiescent stem cells have low rates of bulk translation. Translation increases in activated stem cells and is highest in proliferating progenitors that initiate differentiation. Terminally differentiated cells display low rates of translation. The table shows examples of stem cells in which translation rates follow this pattern: hematopoietic stem cells (HSCs), neural stem cells (NSCs), and hair follicle stem cells (HFSCs). q: quiescent; a: activated.

However, new techniques, known as bio-orthogonal non-canonical amino acid tagging (BONCAT) (Dieterich et al., 2007; Dieterich et al., 2006) and fluorescent non-canonical amino acid tagging (FUNCAT) (Dieterich et al., 2010), have enabled direct visualization of translation in tissue samples and comparison between cell types *in situ*, leading to a different conclusion. In this case, a transient decrease in the overall translation rate was observed as mESCs differentiated into epiblasts, increasing again during neuroectodermal differentiation (Figure 1A) (Corsini et al., 2018). One likely explanation for the discrepancy in translation rates between cultured and *in vivo* mESCs is that protein synthesis is artificially repressed by the factors added to maintain pluripotency in *ex vivo* cultures, leukemia inhibitory factor (LIF) and bone morphogenetic protein 4 (BMP4) (Friend et al., 2015).

In adult stem cells, however, a clearer picture emerges of how the translation rate changes during differentiation. In most stem cell models, translation is low in stem cells, increasing their differentiating progeny, but this increase is reversed when cells terminally differentiate and become postmitotic (Figure 1B).

This was first shown in one of the best characterized adult stem cell populations, hematopoietic stem cells (HSCs), which show low translation rates. As the progeny of HSCs progress along the differentiation pathway through highly proliferative transit-amplifying stages, translation rates increase, remaining high as these progenitors become more lineage-restricted, but eventually dropping in further differentiated cell types (Signer et al., 2014). This latter observation was consistent with previous work showing that the polysome fraction decreased during myeloid differentiation from a promyelocytic cell line in culture, indicating an overall decrease in protein synthesis during terminal differentiation (Krichevsky et al., 1999). Similarly, adult neural stem cells (NSCs) in the sub-ventricular zone have lower translation rates than the neuronal progenitors they give rise to, while differentiation of the latter into postmitotic neuroblasts correlates with decreased levels of protein synthesis (Llorens-Bobadilla et al., 2015; Baser et al., 2019). Hair follicle stem cells (HFSCs) also show a similar pattern of increasing translation rates during differentiation into progenitors, followed by a decrease in terminally differentiated cells (Blanco et al., 2016).

Intriguingly, HSCs, NSCs, and HFSCs can all exist in a quiescent state, in which they do not proliferate; in all cases, quiescent stem cells have significantly lower translation rates than activated stem cells (Signer et al., 2014; Llorens-Bobadilla et al., 2015; Blanco et al., 2016). Although this observation, together with the fact that postmitotic cells tend to have lower translation rates than proliferative progenitors, suggests a link between the translation rate and cell proliferation, proliferation only accounts for part of the difference in protein synthesis rates observed, at least in both blood and hair follicle lineages (Signer et al., 2016; Blanco et al., 2016).

One interesting exception to the general trend that stem cells have lower translation rates than their differentiating offspring is seen in intestinal stem cells (ISCs) in *Drosophila*, which give rise to daughters that are postmitotic, without a transit-amplifying stage (Micchelli and Perrimon, 2006; Ohlstein and Spradling, 2006). Obata et al. (2018) showed that ISCs have the highest bulk translation rate of all cell types in the *Drosophila* intestine, suggesting that differentiating stem cell daughters that immediately become postmitotic does not increase their translation rate, consistent with observations of lower protein synthesis in non-dividing cells in other tissues.

Altogether, these studies indicate that global translation is dynamically regulated during stem cell activation and differentiation and suggest a general pattern (Figure 1B): each step from the activation of quiescent stem cells to differentiation into proliferative progenitors results in an increase in the translation rate. This high rate of protein synthesis is sustained in proliferating progenitors as they continue to mature, until differentiation into postmitotic cells is associated with a decrease in translation. Given this tight control of overall translation rates during development, we focus on the regulation of global protein synthesis in stem cells and their differentiated offspring and how this contributes to gene expression and the maintenance of cell identity.

Several mechanisms have been described to regulate the translation of individual mRNAs, and these include regulating mRNA splicing, stability, and methylation, as well as microRNAs (Jackson et al., 2010; Zhang M. et al., 2020). The *Drosophila* germline provides an excellent example to understand how RNA-binding proteins can affect the translation of key factors regulating self-renewal and differentiation (Slaidina and Lehmann, 2014; Blatt et al., 2020); however, in this review, we will focus on mechanisms that affect global translation rates, in particular translation initiation and ribosome biogenesis, and how they influence stem cell identity.

### Translation Initiation and Its Regulation

Initiation is thought to be the rate-limiting step of protein synthesis, determining which transcripts are translated and how much protein is produced (Duncan et al., 1987; Shah et al., 2013), and it is subjected to regulation by multiple upstream inputs, including nutrient-responsive signals, growth factor signaling, and the amount of ribosomes available in the cell (Sonenberg and Hinnebusch, 2009; Jackson et al., 2010; Roux and Topisirovic, 2018). These regulatory interactions determine both the total rate of translation and the specificity of translated mRNAs. Many signals governing translation rates converge either on controlling the rate of assembly of initiation factors at the m^7^G 5’ cap of the mRNA or the availability of the initiator tRNA carrying methionine (Met-tRNA_i_^Met^).

**FIGURE 2:**
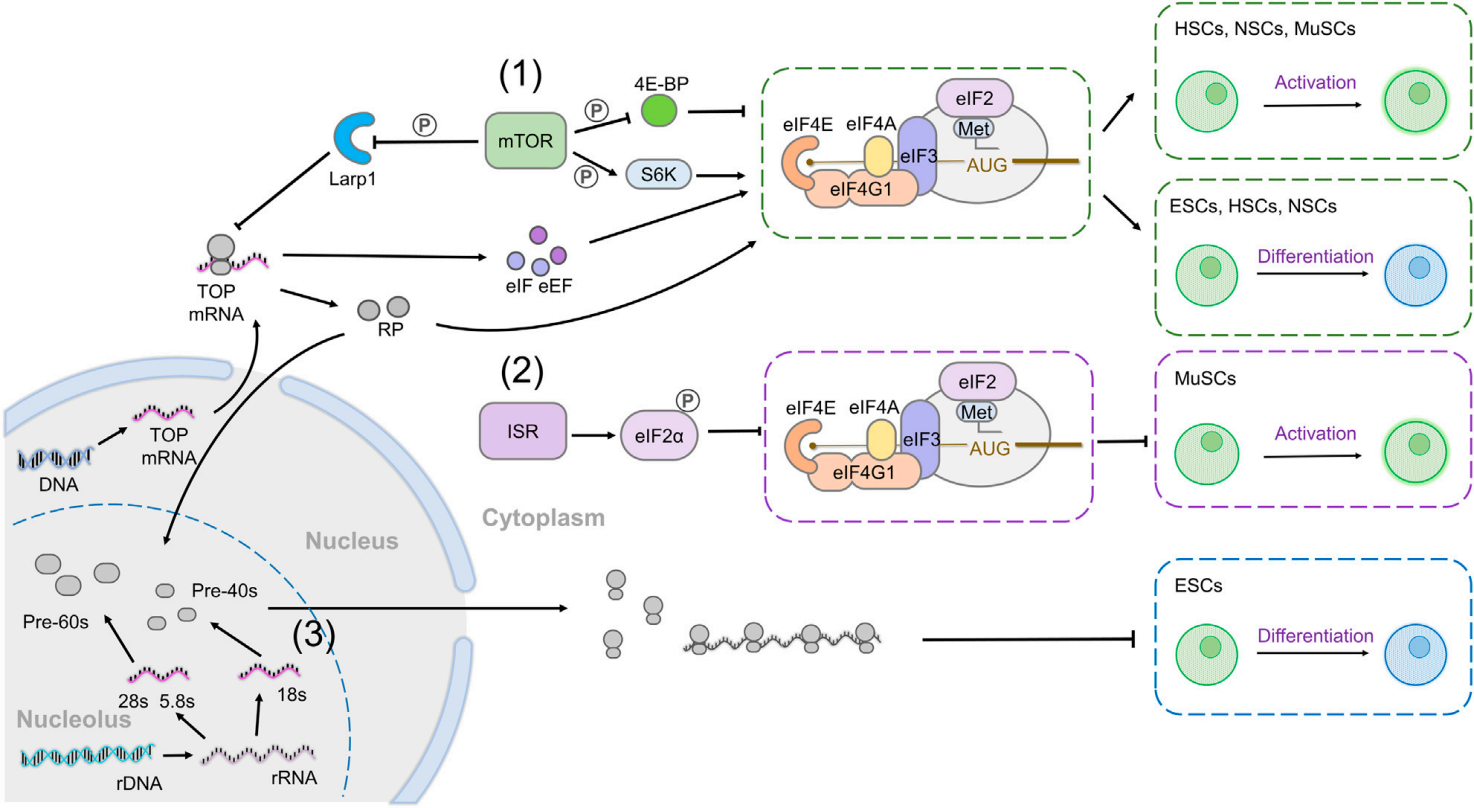
Global mechanisms of translation regulation and their roles in stem cell maintenance. (1) mTOR activity increases global translation through its effectors 4E-BP and S6K and promotes cap-dependent translation. In addition, mTOR increases the translation of TOP mRNAs, which include most components of the translation machinery including translation initiation factors (eIFs), translation elongation factors (eEFs), and ribosomal proteins (RPs). Increased mTOR signaling leads to the activation of HSCs, NSCs, and MuSCs from quiescence and induces differentiation of ESCs, HSCs, and NSCs (green boxes). (2) The integrated stress response (ISR) pathway promotes eIF2ɑ phosphorylation, which reduces eIF2ɑ association with Met-tRNA_i_^Met^ and impairs global translation. P-eIF2ɑ prevents MuSC activation from quiescence (purple box). (3) Ribosomes are assembled in the nucleolus. Decreasing the rate of ribosome biogenesis results in ESC differentiation (blue box).

Canonical translation begins with the eukaryotic initiation factor (eIF) 4E binding to the 5′cap of mRNAs, and assembling a complex known as eIF4F, composed of eIF4E, the helicase eIF4A which unwinds secondary structure and eIF4G. eIF4G acts as a scaffold to bring other initiation complexes, together with the 40S small ribosome subunit to the 5′ end of the mRNA, from where the ribosome will begin scanning for a start codon (Figure 2). Initiation of translation requires Met-tRNA_i_^Met^, which is brought to the ribosome as part of the so-called ternary complex formed of eIF2 bound to GTP and Met-tRNA_i_^Met^.

A major regulator of global cellular translation rates is the mechanistic target of rapamycin (mTOR) pathway (Albert and Hall, 2015; Liu and Sabatini, 2020; Ma and Blenis, 2009), which regulates cell growth and metabolism in response to extracellular growth factors and amino acid levels (Showkat et al., 2014). Two of the best characterized effectors of mTOR are ribosomal protein S6 kinase 1 (S6K1) and eIF4E-binding protein (4E-BP), both of which are phosphorylated upon mTOR activation. 4E-BP binds and sequesters eIF4E, preventing it from interacting with the 5′ mRNA cap, but phosphorylation of 4E-BP by mTOR inactivates it, releasing eIF4E and promoting cap-dependent translation (Figure 2 (1)) (Brunn et al., 1997; Hara et al., 1997; Gingras et al., 1999). Phosphorylated S6K1 increases the activity of several proteins involved in mRNA translation, including eIF4B and ribosomal protein S6 (RpS6), and inactivates translational repressors such as eukaryotic elongation factor-2 kinase (eEF2K), programmed cell death 4 (PDCD4), and La ribonucleoprotein 1 (Larp1) (Ferrari et al., 1991; Wang et al., 2001; Yang et al., 2003; Raught et al., 2004; Fonseca et al., 2015; Hong et al., 2017). Through various targets, activation of mTOR specifically increases the translation of mRNAs containing 5′ terminal oligopyrimidine (TOP) or TOP-like motifs, which consist of a 5’ cytidine at the cap immediately followed by a stretch of 4–15 pyrimidines (Hsieh et al., 2012; Thoreen et al., 2012; Meyuhas and Kahan, 2015; Hong et al., 2017; Iezaki et al., 2018; Philippe et al., 2018; Jia et al., 2021). Interestingly, many mRNAs encoding components of the translation machinery, including but not limited to translation elongation factors (eEFs), some translation initiation factors (eIFs) and most ribosomal proteins, have a TOP motif (Iadevaia et al., 2008; Meyuhas and Kahan, 2015; Hong et al., 2017). Thus, activation of mTOR and its effectors dramatically increases the synthesis of the translation machinery itself, as well as increasing the efficiency of existing translation factors.

Another critical regulator of cellular translation rates is a signaling pathway known as the integrated stress response (ISR). The ISR dramatically decreases mRNA translation following cellular stresses by phosphorylating eIF2ɑ, which prevents its assembly into the ternary complex with GTP and Met-tRNA_i_^Met^ (Figure 2 (2)) (Wek et al., 2006; Pakos-Zebrucka et al., 2016; Costa-Mattioli and Walter, 2020). There are four known kinases that phosphorylate eIF2ɑ in response to various physiological or environmental stresses: PKR-like ER kinase (PERK) is activated downstream of ER stress; general control non-derepressible 2 (GCN2) is responsive to amino acid deprivation; protein kinase RNA-activated (PKR) senses infection-derived dsRNA; and heme-regulated inhibitor (HRI) binds hemin and is disinhibited upon cellular heme deficiency. ISR activation is thought to restore homeostasis and save energy under adverse conditions, particularly by restraining translation; however, a subset of transcripts is specifically translated when eIF2ɑ is phosphorylated, providing a mechanism for the upregulation of stress response genes when most translation is inhibited.

Finally, in addition to these pathways which are dedicated to growth control, other signaling pathways that control patterning during development and influence self-renewal decisions in stem cells can also affect translation. In particular, the Ras/mitogen-activated protein kinase (MAPK) pathway also promotes cellular growth and translation through promoting the activity of eIF4F, *via* its effector MAPK-interacting kinase 1 (Mnk1) (Waskiewicz et al., 1999). In sum, translation initiation is under the control of multiple signaling pathways, enabling the coordination of protein synthesis rates with other inputs into cell identity.

### Ribosomal Biogenesis

Another critical parameter affecting the amount of protein produced in a cell is the number of ribosomes available for translation. Ribosome biogenesis is a complex process bringing together the ribosomal RNAs (rRNAs) and ribosomal proteins into the small and large ribosomal subunits, with the cooperation of non-ribosomal factors, such as small nucleolar ribonucleoproteins (SnoRNPs) (Figure 2 (3)). The amount of rRNA and protein available and the rate of assembly depend on several factors, including cellular stress, nutrient availability, and signaling (de la Cruz et al., 2018; Pelletier et al., 2018; Klinge and Woolford, 2019). The rRNAs are transcribed from nuclear DNA by two specific RNA polymerases (RNA Pol), RNA Pol I, which transcribes most rRNAs and RNA Pol III, which transcribes the 5s rRNA and tRNAs. However, rRNA synthesis and ribosome biogenesis also requires the action of RNA Pol II (Abraham et al., 2020). mTOR has emerged as a critical regulator of ribosome assembly, as its activity coordinately increases the transcription of rRNA and ribosomal proteins. Indeed, mTOR directly regulates the activity of RNA Pol I and RNA Pol III (Powers and Walter, 1999). Similarly, Ras/MAPK signaling increases rRNA synthesis to mediate its effects on growth (Stefanovsky et al., 2001).

Finally, although ribosomes were assumed to be equal and identical, recent work has identified that the composition of ribosomal proteins can change from cell-to-cell, and that, in turn, this composition can affect the mRNAs which are translated (Genuth and Barna, 2018). Thus, both abundance and specificity of ribosomes can be regulated to control overall translation rates and specificity in cells.

## Changes in Bulk Translation Influence Stem Cell Maintenance and Differentiation

### mTOR Promotes Stem Cell Activation and Differentiation Through Increased Translation

mTOR activity changes during stem cell differentiation or activation, and in many cases increased mTOR signaling is sufficient to induce differentiation. The differentiation of *ex vivo*-cultured ESCs, derived from both human and mouse, is coupled with the activation of mTOR activation indicated by phosphorylation of 4E-BP1, RPS6, and eIF4B (Sampath et al., 2008; Easley et al., 2010; Zhou et al., 2020). Similarly, mTOR activity is reduced during the early stages of reprogramming somatic cells into induced pluripotent stem cells (iPSCs) (Wang et al., 2013; Wu et al., 2015). Importantly, while mTOR activity is not required to maintain ESC self-renewal, activating mTOR or its effector S6K primes ESCs to differentiate and mTOR hyperactivity prevents the reprogramming of somatic cells into iPSCs (Murakami et al., 2004; Easley et al., 2010; He et al., 2012; Wang et al., 2013; Wu et al., 2015). Reprogramming also requires the presence of 4E-BPs (Tahmasebi et al., 2014), further implicating translation as one of the key cellular processes by which mTOR activity leads to the loss of pluripotency (Figure 2 (1)).

Similarly, increasing mTOR activity is sufficient to promote differentiation and loss of self-renewal ability in a variety of adult stem cell types across organisms, from HSCs and NSCs in mouse to *Drosophila* intestinal stem cells and both somatic and germline stem cells in the gonads (Zhang et al., 2006; Chen et al., 2009; Sun et al., 2010; Magri et al., 2011; Kapuria et al., 2012; Quan et al., 2013; Yuen et al., 2021). In HSCs, the deletion of *Pten*, a repressor of mTOR, or constitutive activation of mTOR, lead to ectopic proliferation of transit-amplifying progenitors, resulting in leukemia. However, despite this over-proliferation, increases in mTOR activity result in a depletion of HSCs, as determined by a deficiency in reconstituting the blood lineage upon transplantation into an immunodeficient host (Yilmaz et al., 2006; Zhang et al., 2006; Guo et al., 2008; Chen et al., 2009; Magee et al., 2012). In elegant genetic experiments, Signer et al. (2014) showed that *Pten* mutant HSCs had higher translation rates than control, and, importantly, that introducing a mutant copy of the *belly spot and tail* (Ferretti et al., 2017), encoding the ribosomal protein Rpl24, could decrease overall translation and restore the self-renewal and reconstitutive capacity of *Pten* mutant HSCs. Further work identified 4E-BP1 and 4E-BP2 as mediators of translational repression in HSCs, and their loss results in a similar decrease in long-term self-renewal ability to that of *Pten* mutant HSCs (Signer et al., 2016). Thus, an increased translation downstream of mTOR activation results in the loss of quiescence, increased proliferation, and eventual loss of the stem cell pool (Figure 2 (1)).

mTOR plays similar roles in regulating NSC quiescence and differentiation, in two different NSC populations, in the sub-ventricular zone (SVZ) of the lateral ventricle and the dentate gyrus. In both postnatal and adult SVZ NSCs, activating mTOR through loss of function of the negative regulators *Pten* or *TSC1*, or gain of function of the activator Rheb, led to increased production of neurons, at the expense of stem cell maintenance (Groszer et al., 2006; Gregorian et al., 2009; Magri et al., 2011; Hartman et al., 2013; Mahoney et al., 2016). Loss of NSCs was attributed to an increased frequency of symmetric divisions generating two proliferative progenitors, rather than self-renewing asymmetric divisions. Similarly, in the dentate gyrus, *Pten* loss mobilizes quiescent NSCs and induces them to proliferate through symmetric self-renewing divisions, but eventually results in increased terminal differentiation and stem cell loss (Bonaguidi et al., 2011). As in HSCs, 4E-BP2 is a critical downstream effector of mTORC1, controlling cap-dependent translation during neuronal differentiation in the SVZ (Hartman et al., 2013; Mahoney et al., 2016) (Figure 2 (1)).

The function of mTOR in controlling exit from quiescence is remarkably conserved across tissues and even species. Indeed, TOR activation in quiescent *Drosophila* NSCs arrested in either G_0_ or G_2_ leads to cell cycle entry and differentiation (Chell and Brand, 2010; Sousa-Nunes et al., 2011; Otsuki and Brand, 2018). In muscle stem cells (MuSCs, also known as satellite cells), mTOR activity promotes an “alert” state of quiescence in which cells are primed for reactivation, leading them to re-enter the cell cycle upon injury or stress (Rodgers et al., 2014). Moreover, in the intestine of both flies and mice, mTOR controls the ability of the population of quiescent stem cells to contribute to the regenerative response following fasting and refeeding (Richmond et al., 2015); however, repeated regenerative episodes and bouts of mTOR activity lead to eventual loss of stem cell maintenance (Haller et al., 2017). In sum, the mTOR pathway is widely associated with stem cell activation and differentiation, and persistent activation leads to the loss of self-renewing potential. In several instances, the effects of mTOR are mediated through its effects on translation through its effectors, 4E-BP, and S6K (Figure 2 (1)).

Of note, although the global translation rate correlates with mTOR activity throughout differentiation in the neural lineage, this is not true in all tissues (Paliouras et al., 2012; Cloetta et al., 2013; Baser et al., 2019). In myeloid progenitors derived from HSCs, mTOR is degraded by the proteasome, yet translation is still regulated by the mTOR target 4E-BP1 (Spevak et al., 2020). In this case, the cell cycle-dependent kinase CDK1 phosphorylates 4E-BP1 to promote eIF4E-dependent translation and maintain high translation rates. Thus, mTOR activity does not always linearly correlate with the overall translation rate and many other regulators may independently regulate translation initiation factors to achieve a precise rate of global translation.

### eIF2α Phosphorylation in Stem Cell Maintenance

eIF2ɑ phosphorylation has also emerged as an important regulator of stem cell maintenance through effects on global translation. High levels of p-eIF2ɑ are observed in both *ex vivo*-cultured mESCs and murine MuSCs (Friend et al., 2015; Zismanov et al., 2016). Although in mESCs, there are conflicting data as to whether p-eIF2ɑ levels decrease with differentiation, the signaling factors BMP4 and LIF, which maintain pluripotency in ESCs, both increase eIF2ɑ phosphorylation (Friend et al., 2015). Indeed, preventing dephosphorylation of eIF2ɑ is sufficient to prevent differentiation even in the absence of LIF. Similarly, in intestinal stem cells in *Drosophila*, eIF2ɑ is phosphorylated by PERK in response to ER stress, and promotes stem cell proliferation. Continued eIF2ɑ phosphorylation results in tissue dysplasia with an accumulation of undifferentiated cells, consistent with a role for p-eIF2 in maintaining stem cell identity (Wang et al., 2015).

In MuSCs, eIF2ɑ is highly phosphorylated in quiescent cells. Indeed, replacing endogenous eIF2ɑ with a non-phosphorylatable mutant, results in the short-term activation of quiescent stem cells, increased translation and proliferation, and myogenic differentiation. In the long term, however, MuSCs unable to phosphorylate eIF2ɑ are lost from the stem cell population (Zismanov et al., 2016) (Figure 2 (2)). Thus, in MuSCs at least, eIF2 is a critical regulator of both overall translation rates and quiescence, precise regulation of which is essential to maintain long-term self-renewal potential.

### Ribosome Biogenesis Is Highly Regulated and Required for Stem Cell Maintenance

Given the importance of translation in regulating stem cell biology, ribosome biogenesis has also emerged as a critical factor controlling self-renewal and differentiation. In hematopoietic and muscle lineages, rRNA transcription follows a similar pattern to the bulk translation rate, increasing during the differentiation of stem cells into proliferative progenitor cells and decreasing in terminally differentiated cells (Larson et al., 1993; Hayashi et al., 2014; Stedman et al., 2015; Gayraud-Morel et al., 2018). In addition to rRNA, the expression of regulators controlling rRNA transcription or maturation also correlates with the bulk translation rate during stem cell differentiation. In zebrafish, the expression of *ddx27*, encoding a regulator of rRNA maturation, is detected in activated MuSCs and proliferating myoblasts, and decreases when cells terminally differentiate (Bennett et al., 2018). In mESCs and hESCs, the expression of rRNA and the regulators of ribosome biogenesis correlate with the overall translation rate both *in vivo* and *in vitro* (Watanabe-Susaki et al., 2014; Zaidi et al., 2016; Corsini et al., 2018).

Despite this broad correlation, it is notable that ribosome biogenesis is proportionally higher in stem cells than that in differentiating cells, relative to the rate of translation (Stedman et al., 2015; Zaidi et al., 2016; Gayraud-Morel et al., 2018). Strikingly, in the *Drosophila* germline, rRNA transcription is the highest in germline stem cells (GSCs), while translation is lower in GSCs than in differentiated offspring (Zhang et al., 2014; Sanchez et al., 2016). These observations suggest a specific requirement for increased ribosome biogenesis in stem cells.

Indeed, disrupting ribosomal biogenesis in many stem cell models leads to defects in both survival and self-renewal (Stedman et al., 2015; Sanchez et al., 2016; Bennett et al., 2018; Baral et al., 2020; Farooq et al., 2020; Saez et al., 2020), while fully differentiated somatic cells demonstrate less dependency on ribosome biogenesis (Bennett et al., 2018; Gayraud-Morel et al., 2018; Saez et al., 2020). Impairing rRNA transcription induces differentiation in *Drosophila* GSCs and mouse hematopoietic progenitor cells (Hayashi et al., 2014; Zhang et al., 2014). Importantly, this effect on hematopoiesis is not mediated by a global repression of translation or a cell cycle arrest as inhibiting overall protein synthesis by cycloheximide and puromycin, or inhibiting cell cycle by roscovitine, a CDK inhibitor, does not have the same effect (Pilz et al., 1987; Hayashi et al., 2014). Similarly, disrupting ribosomal biogenesis in mESCs or hESCs by either repressing rRNA maturation or transcription triggers the expression of differentiation-related genes, and this is coupled with a reduced expression of pluripotent mRNAs such as OCT4 or SOX2 (You et al., 2015; Woolnough et al., 2016; Zhang H. et al., 2020). Importantly, the overexpression of fibrillarin, an important regulator of ribosomal RNA processing, can sustain pluripotency in the absence of LIF(Watanabe-Susaki et al., 2014) (Figure 2 (3)).

Altogether, ribosomal biogenesis has begun to be recognized as a major factor maintaining pluripotency and self-renewal potential. Little work to date has sought to identify the upstream regulators ensuring the coordinated production of high levels of rRNA, ribosomal proteins, and assembly factors in stem cells. Nonetheless, it is clear that elevated ribosome levels are required to maintain stem cell potential. Together with strong evidence showing lower translation in stem cells, this suggests that ribosome levels and translation rates are not correlated in stem cells; one suggestion is that a large pool of ribosomes is required to prepare cells to rapidly increase their translation rates and change their proteome during differentiation (Saba et al., 2021). However, this is hard to reconcile with the fact that decreasing ribosome biogenesis promotes differentiation, indicating that either ribosome biogenesis itself or the availability of large numbers of ribosomes relative to the amount of transcripts is in itself important for stem cell biology.

## From Global Translational Control to Specific Protein Expression: Mechanisms Ensuring Selectivity in Translation in Stem Cells

How do changes in global translation rates or ribosome biogenesis affect stem cell maintenance? At least in part, the answer to this question lies in the selective translation of specific transcripts in response to changes that globally alter translation rates. Indeed, accumulating evidence show that specific mRNAs are translated in stem or differentiated cells, without always being accompanied by changes in mRNA abundance (Unwin et al., 2006; Lu et al., 2009; Habowski et al., 2020). In other words, mRNA translation is a regulatory mechanism allowing gene expression changes independently of transcription.

### Specific Targets of mTOR Activity

**FIGURE 3:**
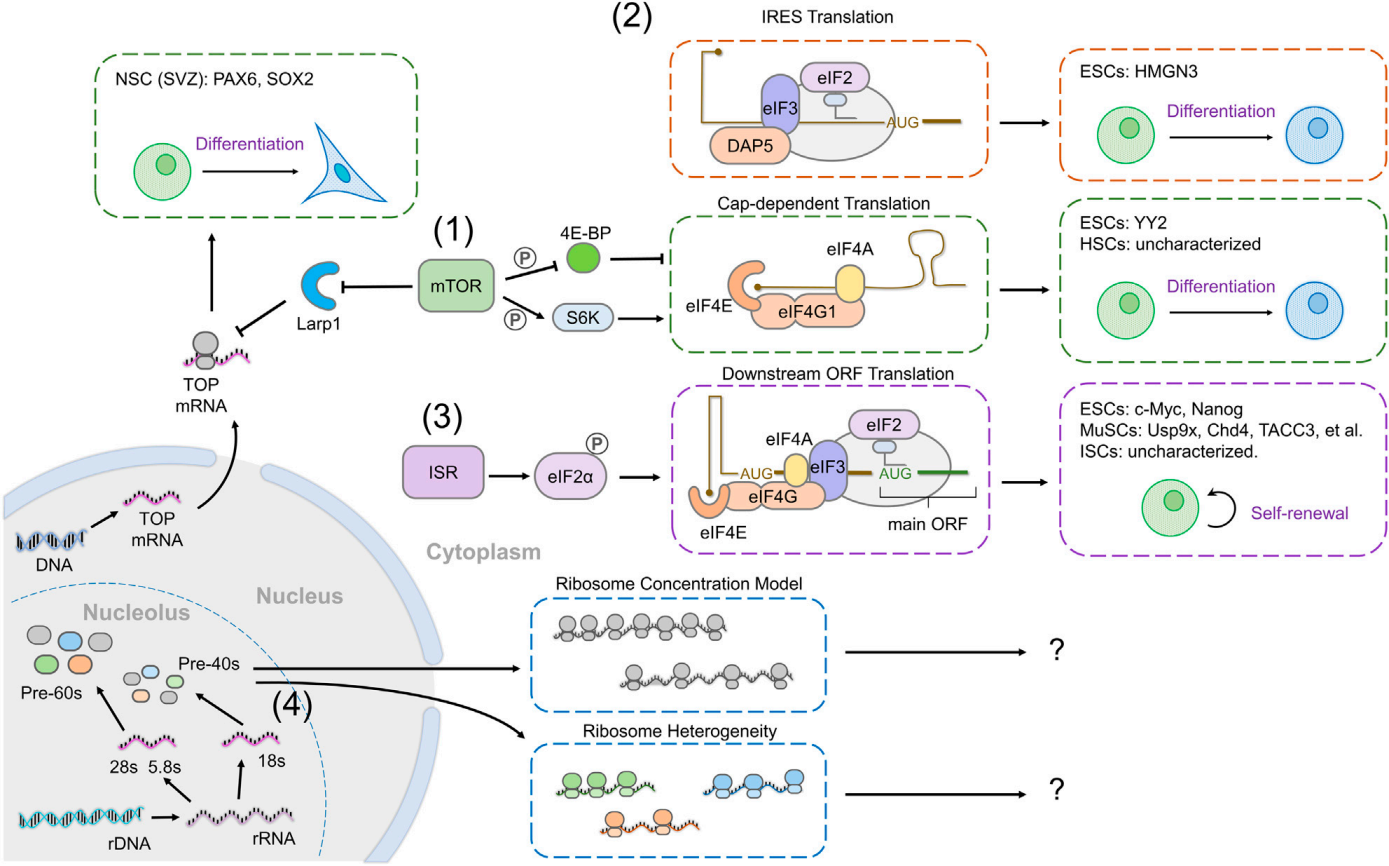
Global translation mechanisms result in selective translation of specific transcripts to regulate stem cell fate. (1) Increased cap-dependent translation downstream of mTOR activity can result in selective translation of transcripts that have low translation efficiency. In ESCs, the mRNA encoding YY2 is one such target, in other tissues (HSCs), whether mTOR activity results in specific target expression is unknown (green box). mTOR also promotes the translation of mRNAs with TOP-like motifs, such as PAX6 and SOX2, which are translationally repressed during neuronal differentiation, in response to reduced mTOR activity. (2) Internal ribosome entry site (IRES)-mediated translation occurs when cap-binding is inhibited and can direct transcript-specific translation. In ESCs, DAP5 replaces eIF4G to promote IRES-dependent translation and ESC differentiation, through translation of HMGN3 (orange boxes). (3) p-eIF2ɑ selectively promotes the translation of mRNAs with an upstream open reading frame (uORF). In ESCs, p-eIF2ɑ promotes the translation of the mRNAs encoding c-Myc and Nanog. In MuSCs, p-eIF2ɑ promotes the translation of mRNAs coding for Usp9x, Chd4, TACC3, etc. (purple box). (4) Ribosomes can selectively regulate mRNA translation. Two models have been proposed, the ribosome concentration model in which the amount of ribosomes available affects the translation of transcripts with high or low translation efficiency differently, or ribosome heterogeneity in which different ribosomal subunit composition directs specific translation of particular transcripts (blue boxes). Although ribosomes impact stem cell fate, no evidence directly supports either model to date.

Although mTOR activity increases bulk translation by increasing the activity of initiation complexes, it disproportionally targets mRNAs containing TOP motifs for increased translation (Hsieh et al., 2012; Thoreen et al., 2012; Meyuhas and Kahan, 2015; Hong et al., 2017; Iezaki et al., 2018; Philippe et al., 2018; Jia et al., 2021). For instance, during the differentiation of SVZ neurogenic progenitors into neurons, both mTOR activity and bulk translation levels decrease (Baser et al., 2019). Notably, transcripts containing a pyrimidine-rich motif, similar to the TOP motif, are specifically repressed during differentiation; these encode both ribosomal proteins and transcription factors regulating stem cell identity such as Pax6 and Sox2, providing a mechanism by which mTOR activity correlates both with translation rates and with fate acquisition (Baser et al., 2019) (Figure 3 (1)).

### eIF4F-Mediated Cap-Dependent Translation and Non-Canonical Translation

Regulation of the activity and ability of the eIF4F complex to bind the mRNA cap also provides a means to achieve specificity in translation (Hernández et al., 2020). 4E-BP is a major regulator of eIF4F activity, and its regulation by mTOR and other signals makes it an ideal modulator to act as a switch for gene expression. Surprisingly, in mESCs, loss of function of *4E-BP1/2* does not influence the global translation rate, but results in the loss of pluripotency marker expression (Tahmasebi et al., 2016). This effect is mediated by the selective translation of Yin Yang 2 (YY2) upon ablation of 4E-BP. The *YY2* mRNA retains an intron in its 5’ UTR, making its translation acutely sensitive to eIF4E activity due to a complex secondary structure (Figure 3 (1)).

Other regulators of the assembly of the eIF4F complex also contribute to specific gene expression. In SVZ neural precursors, 4E-T competes with eIF4G for binding to eIF4E1, forming a complex which represses the translation of neurogenic mRNAs (Yang et al., 2014). Knock down of eIF4E1 or 4E-T promotes precursor differentiation while knocking down eIF4G1, on the contrary, impairs differentiation, indicating that in the SVZ, the main function of eIF4E1 in neural precursors is to repress the translation of neurogenic mRNAs.

Another regulator of eIF4F function is eIF4G2 (also named death-associated protein 5 (DAP5) or the novel APOBEC1 target 1 (NAT1)). eIF4G2 contains a similar C-terminal region to eIF4G1, enabling it to interact with eIF3 and eIF4A, but lacks an N-terminal eIF4E-binding domain, meaning that eIF4G2 promotes translation independently of eIF4F, and instead stimulates the translation of mRNAs containing an element known as an internal ribosome entry site (IRES) (Henis-Korenblit et al., 2002). DAP5 is required for neural and mesodermal differentiation of hESCs (Yoffe et al., 2016). The block in differentiation observed upon DAP5 depletion is not the consequence of a global translational repression, but instead it is due to selective IRES-driven translation by DAP5, in particular of the chromatin modifier HMGN3. Similarly, NAT1, the mouse homolog of DAP5, is required for the differentiation of mESCs (Sugiyama et al., 2017; Yamanaka et al., 2000). This was ascribed to NAT1 promoting the translation of two components of the ERK signaling pathway, which is required for ESC differentiation (Figure 3 (2)). However, the role of DAP5 in ESCs is still not fully understood, and may differ between mouse and humans, as loss of DAP5 in primed mESCs results in reduced self-renewal and defects in neural differentiation, in contrast to loss of DAP5 in naïve mESCs, which prevents differentiation into all cell types (Takahashi et al., 2020).

Thus, the eIF4F complex is a central node through which multiple regulators can control bulk protein synthesis and the translation of specific subsets of mRNAs. Indeed, due to the presence of the eIF4A helicase in the eIF4F complex, mRNAs with long and/or complex secondary structures are particularly sensitive to eIF4F activity. Thus, changes in eIF4F activity (in the absence of some of the more specific regulations described earlier) can result in a binary regulation of individual mRNA translation (Leppek et al., 2018), enabling the fine control of gene expression. It is highly likely that in other situations where bulk translation is increased during stem cell differentiation, such as in HSCs, the effects of translation increase on cell identity are mediated by such mechanisms.

### eIF2α-p Selectively Regulates mRNAs With uORFs

Although eIF2ɑ phosphorylation dramatically reduces bulk translation, a subset of mRNAs is translated under these conditions (Baird et al., 2014). The best characterized example is the translation of the mRNA encoding ATF4 (Vattem and Wek, 2004; Asano, 2021), which contains two upstream open reading frames (uORFs), preventing the translation of the main open reading frame. Phosphorylation of eIF2 delays re-initiation of translation at the second uORF, resulting in initiation and translation at the main ATF-coding open reading frame.

Ribosome profiling in mESCs has revealed higher translation of uORFs in ESCs than EBs (Ingolia et al., 2011). Intriguingly, transcripts encoding the pluripotency factors, c-*Myc* and *Nanog*, have multiple uORFs (Figure 3 (3)). Whether this change in uORF translation during ESC differentiation is related to eIF2 activity, and whether it plays a role in fate determination is yet to be established.

A more direct example of p-eIF2ɑ-dependent expression of specific transcripts is seen in MuSCs, in which quiescence and self-renewal depend on eIF2ɑ phosphorylation (Zismanov et al., 2016). A study of proteins upregulated by eIF2 phosphorylation without accompanying changes in mRNA levels identified several genes encoding mitotic spindle assembly factors, in particular *TACC3.* The *TACC3* transcript contains multiple uORFs and the protein is present in stem cells but downregulated in differentiating myoblasts. Importantly, TACC3 is required for MuSC expansion and self-renewal, demonstrating the functional importance of selective translation of uORF-containing transcripts in stem cell maintenance (Vattem and Wek, 2004; Fujita et al., 2021) (Figure 3 (3)).

### Translational Specificity From Ribosomes: Effects of Ribosome Concentration and Subunit Composition

Stem cells require high levels of ribosome biogenesis for maintenance, despite lower translation rates, raising the possibility that ribosome numbers may play a role in specifically regulating stem cell gene expression. One model put forward to explain this is that different transcripts are differentially sensitive to ribosome concentration; mRNAs that are less efficiently translated would require a higher concentration of ribosomes to be expressed (Gabut et al., 2020; Lodish, 1974; Mills and Green, 2017) (Figure 3 (4)). Evidence in support of this model has been found in the case of a mutation in a ribosomal protein chaperone that causes Diamond-Blackfan anemia, which leads to reduced ribosome numbers but specifically alters the translation of a susbset of transcripts (Khajuria et al., 2018). This study linked a lineage commitment decision in progenitors with ribosome levels for the first time, but as yet, the same findings have not been reproduced in a stem cell model. Future work will determine whether this model does indeed apply to stem cells, and importantly, what determines the sensitivity of particular mRNAs to ribosome concentration.

Another means by which specificity in transcript translation can be achieved by ribosomes is through the specific subunit composition of each ribosome (Figure 3 (4)). Although ribosomes were initially assumed to be equivalent and to translate all mRNAs equally, work in the past decade has established that different ribosomes incorporate different ribosomal proteins. Different ribosomal proteins can confer mRNA sequence recognition (Genuth and Barna, 2018) and direct specific translation through IRES-dependent mechanisms. Intriguingly, mESCs display different ribosome subunit stoichiometries in monosomes and polysomes, and these associate with different mRNAs (Shi et al., 2017). Recent work in the *Drosophila* germline has shown that a paralogue of RpS5 is required for normal progression of differentiation and preferentially promotes translation of a subset of transcripts (Kong et al., 2019; Jang et al., 2021). These tantalizing observations raise the possibility that different incorporation of ribosomal subunits into ribosomes may regulate stem cell behavior; however, this has not yet been demonstrated.

## Conclusions and Perspectives

From an initial view of mRNA translation as a “housekeeping” function that is performed equally in all cells and for all transcripts, our understanding has evolved to grasp the complexity and precision of translational regulation and its ability to tune cell fate. This is especially evident in stem cells where the decision to self-renew and differentiate is exquisitely sensitive to changes in protein synthesis. This raises the important question as to why translational regulation is such a pervasive mechanism to control identity across stem cells. One possible explanation is that stem cell differentiation requires a large remodeling of the cell’s proteome. Indeed, another important cellular function in stem cell biology is protein degradation, emphasizing the importance of accurate regulation of the cellular protein content in stem cell fate decisions (Llamas et al., 2020). Additionally, transcription is an inherently noisy process (Elowitz et al., 2002; Raj et al., 2010; Raser and O'Shea, 2005); this noise may play important roles in enabling cell decisions (Eldar and Elowitz, 2010). However, overlaying selective translation onto noisy gene expression could be a way to ensure that cells with the potential to adopt two different fates can only commit to one of these.

As our ability to probe translation increases, it is becoming more apparent that regulatory mechanisms-controlling global translation do not affect all transcripts equally; translation efficiency varies for individual mRNAs in different conditions. Thus, whether bulk translation changes are relevant to stem cell differentiation, or whether all the effects of changes in translation are mediated by the altered translation of a few key transcripts is still an open question.

In addition to contributing to our understanding of the mechanisms underlying self-renewal and differentiation and to our ability to manipulate those processes, and studying translation in stem cells will yield important advances in the study of aging. Reducing mTOR activity has long been known to extend the lifespan and promote continued health of organisms (Liu and Sabatini, 2020). Although other targets of mTOR have been implicated, S6K or eIF4E reduction, or 4E-BP overexpression, can contribute to lifespan extension, suggesting that decreased translation rates are at least partly responsible (Hansen et al., 2007; Syntichaki et al., 2007; Selman et al., 2009; Zid et al., 2009). Moreover, recent work has shown that both RNA Pol I and RNA Pol III, which synthesize rRNAs, mediate lifespan control downstream of mTOR, and that, in *Drosophila*, they exert their effects on lifespan specifically in intestinal stem cells (Filer et al., 2017; Martinez Corrales et al., 2020). As we deepen our understanding of how translational regulation influences stem cell behavior, new avenues for interventions that mitigate the effects of aging will be opened up.
